# Temporal pitch matching with bilateral cochlear implants

**DOI:** 10.1121/10.0025507

**Published:** 2024-04-01

**Authors:** Justin M. Aronoff, Simin Soleimanifar, Prajna BK

**Affiliations:** Speech and Hearing Science Department, University of Illinois at Urbana-Champaign, 901 South 6th Street, Champaign, Illinois 61820, USA jaronoff@illinois.edu, simins2@illinois.edu, prajnab2@illinois.edu

## Abstract

Interaural pitch matching is a common task used with bilateral cochlear implant (CI) users, although studies measuring this have largely focused on place-based pitch matches. Temporal-based pitch also plays an important role in CI users' perception, but interaural temporal-based pitch matching has not been well characterized for CI users. To investigate this, bilateral CI users were asked to match amplitude modulation frequencies of stimulation across ears. Comparisons were made to previous place-based pitch matching data that were collected using similar procedures. The results indicate that temporal-based pitch matching is particularly sensitive to the choice of reference ear.

## 1. Introduction

A number of studies have investigated bilateral cochlear implant (CI) users' interaural pitch mismatches (Aronoff *et al.*, 2016, 2019; Kan *et al.*, 2013; Reiss *et al.*, 2008, 2007), highlighting differences in perceived pitch across ears. Although many studies have focused on the interaural mismatch of place-based pitch, temporal-based pitch is also important for CI users, especially for vocal pitch and music perception (Chatterjee and Peng, 2008; Green *et al.*, 2005; Laneau *et al.*, 2006). Although some studies, like Pijl (1997), show minimal interaction between rate discrimination (temporal information) and place of stimulation differences (place information), others highlight the combined impact of temporal and place cues for pitch perception for CI users (Bissmeyer and Goldsworthy, 2022; Landsberger *et al.*, 2016; Luo *et al.*, 2012; McKay *et al.*, 2000). As such, understanding the effects of place-mismatches likely requires also understanding their impact on temporal-based pitch matching. However, measuring interaural temporal-based pitch matching presents challenges.

One of the main challenges of measuring interaural temporal-based pitch mismatches is that CI users have an extremely small range of usable temporal-based pitch, often limited to less than 300 Hz (Ihlefeld *et al.*, 2015; Kong *et al.*, 2009; Kong and Carlyon, 2010). As such, interaural temporal-based pitch mismatches are expected to be difficult to measure (i.e., result in large but random pitch mismatches) for temporal-based pitches near or above 300 Hz, consistent with similar challenges with within-ear rate matching and rate discrimination (Cosentino *et al.*, 2016; Pijl, 1997). The goal of this study was to investigate the properties of temporal-based pitch matching in the best-case scenario where stimulation is place-matched, using amplitude modulated stimuli, investigating the effect of the reference amplitude modulation (AM) frequency, response biases, and reference ear effects. To distinguish effects of temporal-based pitch matching from artifacts of the pitch matching procedure, the properties of the temporal-based pitch matching will be compared to those of previously collected place-based pitch matching data from Aronoff *et al.* (2016).

## 2. Methods

### 2.1 Participants

Twelve bilateral CI users participated in this experiment. All had Cochlear Americas brand devices (Lone Tree, CO). See Table 1 for participant details.

**Table 1.** Participant characteristics.

Subject	Gender	Age onset of hearing loss (years)	Duration of CI use (years)	Age (years)	Place-based pitch matched electrode^a^
I12	Female	Birth (L&R)	9 (L), 7 (R)	48	15
I64	Female	45 (L&R)	10 (L), 8 (R)	62	13
I65	Female	56 (L&R)	6 (L), 7 (R)	67	10
I67	Female	59 (L&R)	2 (L), 5 (R)	70	12
I69	Male	51 (L&R)	3 (L&R)	62	9
I70	Male	42 (L&R)	12 (L), 11 (R)	58	19
I71	Female	5 (L&R)	10 (L), 11 (R)	21	12
I73	Female	50 (L&R)	10 (L&R)	67	16
I74	Male	Birth (L&R)	16 (L), 14(R)	60	13
I76	Female	2 (L), 3 (R)	22 (L), 13 (R)	50	11
I77	Female	3–4 years (L&R)	5 (L&R)	61	11
I78	Female	40 years (L&R)	14 (L), 13 (R)	65	11

^a^ All participants used electrode 11 as the reference electrode except for I71, who used electrode 17 as the reference electrode.

### 2.2 Procedures

Prior to testing, appropriate stimulation levels were determined and stimulation was loudness balanced for each participant, followed by place-based pitch matching. Place-based pitch matching was used to select the electrode pairs for the temporal-based pitch matching task to minimize the effect of place-based pitch mismatches on that task. The procedures for each of these are described in detail below.

#### 2.2.1 Stimulation parameters

Stimulation was controlled by the Nucleus Implant Communicator (NIC3 or NIC4). For those tested with NIC3 (all participants except I69 and I71), stimulation was delivered using the RF Generator (Cochlear, Ltd., Sydney, Australia). For those tested with NIC4, stimulation was delivered using the Cochlear CP910 processor. Stimulation consisted of monopolar stimulations with a phase duration of 32 *μ*s and an interphase gap of 8 *μ*s. A pulse rate of 1000 pulse per second was used, with 500 ms pulse trains. The 1 kHz pulse-rate was chosen because it is above the rate at which CI users can typically utilize temporal information derived from the pulsatile carrier (Kong *et al.*, 2009; Kong and Carlyon, 2010). Threshold (T) and maximum comfort (C) levels were measured for electrode 11, typically in the right ear. This ear served as the reference ear for the place-based pitch matching task described below. If there were deactivated electrodes in the left ear, the left ear was used instead so that participants had more activated electrodes in the target ear for the place-based pitch matching task, given that only one electrode was used in the reference ear for that task, but all electrodes in the target ear were used when possible.

#### 2.2.2 Loudness balancing

After measuring the T and C levels, stimulation was loudness-balanced within ear for all electrodes in the place-based pitch matching target ear not deactivated in the participant's clinical map. This was done at the most comfortable loudness (MCL) level. The percent of the dynamic range (DR; i.e., the range of stimulation intensities extending from T to C levels) that yielded the MCL level was determined, typically for the most apical electrode, which served as a reference for within-ear loudness balancing. For the target ear, the participant was presented stimulation on four adjacent electrodes in sequence at the same percent DR that yielded MCL and asked to indicate if any were louder or softer than the rest. C levels were adjusted until all four electrodes were perceived to be equally loud. Then the next set of four electrodes were selected, using one electrode from the first group plus three adjacent electrodes (e.g., first group of four: electrodes 22, 21, 20, 19; second group of four: electrodes 19, 18, 17, 16). This process was repeated until all electrodes within the target ear were loudness balanced.

Loudness was next balanced across ears by stimulating one electrode in one ear (typically electrode 11) at the percent DR corresponding to MCL followed by stimulating an electrode in the other ear (typically electrode 11). The percent DR used for stimulation was adjusted to find the percent DR for each ear that yielded stimulation that was both at the MCL and loudness balanced. Those percent DRs were used for the place-based pitch matching task, with 0.5 dB roving to minimize potential loudness cues.

#### 2.2.3 Place-based pitch matching

To minimize the confound of place-pitch mismatches when measuring temporal-based pitch matches, place-based pitch matched electrodes were used. One electrode (typically electrode 11) in the reference ear was stimulated, followed by stimulation in the target ear. One participant (I71) had electrodes 1–12 deactivated because they were extracochlear. For that participant, electrode 17 was used as the reference electrode since its frequency allocation was similar to that for electrode 11 in a typical map. The initial electrode stimulated in the place-based pitch matching target ear was selected randomly for each trial. Stimulation in the place-pitch target and reference ear was separated by approximately 500 ms.

Participants controlled the place of stimulation in the target ear using a dial (Powermate; Griffin Technology, Nashville, TN). Turning the dial clockwise changed the target electrode to a more basal electrode and turning the dial counterclockwise changed the target electrode to a more apical electrode. The location of the target electrode was indicated on a slider on a computer screen that changed with movement of the dial. Participants could also choose to use a mouse to move the slider rather than using the dial. After participants moved the dial/slider, they pressed the space bar to hear the reference ear stimulation followed by the target ear stimulation. Participants could repeat this process of moving the dial or slider and listening to the resulting comparison until they perceived the pitch in the two ears to be matched. They indicated this by clicking a button labeled “Done” on the screen. Participants typically completed this pitch matching three times and the place-based pitch matched electrode in the target ear was defined as the median (rounded to the nearest electrode) of their place-based pitch matches and used in the temporal-based pitch matching task.

#### 2.2.4 Temporal-based pitch matching

Temporal-based pitch matching used the same interface and procedure as place-based pitch matching. The reference ear varied across trials, with a block of trials using one ear as the reference followed by a block of trials using the other ear as the reference, with the order of these blocks randomized across participants. Stimulation consisted of 1000 Hz pulse trains, sinusoidally modulated with 100% modulation depth (ranging from T-levels to C-levels). The modulation frequency for the reference ear varied across trials, and consisted of 75, 100, 125, 150, 175, 200, 225, 250, or 275 Hz. Participants could manipulate the modulation frequency for the target ear by using the dial or the slider, covering a range from 50 to 300 Hz in 5 Hz steps, with clockwise turns of the dial increasing the modulation frequency and counterclockwise turns decreasing the modulation frequency. All reference modulation frequencies were used for each reference ear, typically repeated twice. For participants whose first and second temporal-based pitch matches differed by 50 Hz or more, a third trial was conducted when time allowed. The temporal-based pitch match was defined as the median temporal-based pitch match per reference modulation frequency and ear.

## 3. Results

### 3.1 Effect of reference AM frequency

**Fig. 1.**
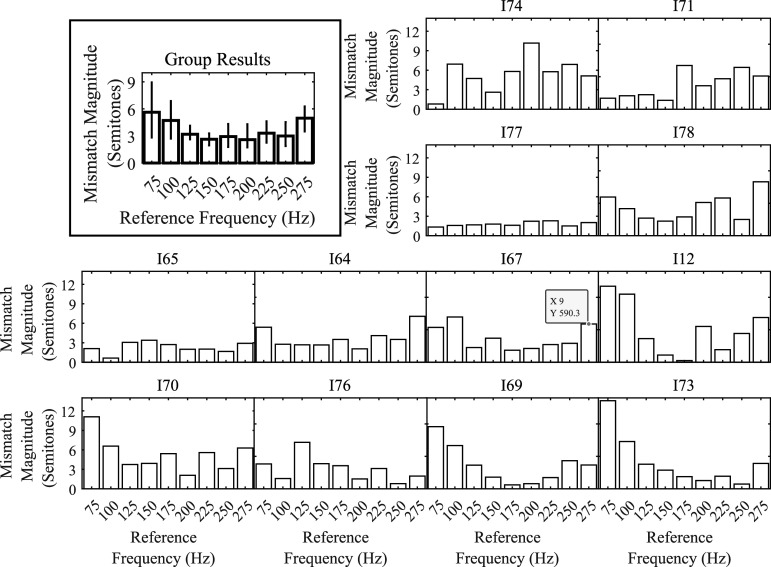
Individual results showing the median mismatch magnitude (unsigned difference between reference and target frequency) per reference frequency per participant, pooled across ears. The participants are ordered based on the ratio of the mismatch magnitude for the 75 Hz reference frequency to the mismatch magnitude for the 275 Hz reference frequency. The inset contains group results showing the 20% trimmed mean of the mismatch magnitude and 95% confidence interval for each target frequency, pooled across reference ear and participants.

All temporal-based pitch matches were converted to semitones (an octave is 12 semitones) for analysis. To examine the hypothesis that pitch mismatches are difficult to measure for temporal-based pitches near 300 Hz and to examine if there are other limitations on measuring temporal-based pitch judgments, the median absolute value of the difference between the reference and target AM frequency (i.e., the mismatch magnitude), pooled across ears and participants, was calculated for three frequency ranges: low (75–125 Hz), middle (150–200 Hz), and high (225–275 Hz). A bootstrap repeated-measures ANOVA was conducted based on the 20% trimmed means. There was no significant effect of frequency range (*p* > 0.05; see Fig. 1). Pooling across frequency range, the 20% trimmed mean of the mismatch magnitude was 4.5 semitones (95% confidence interval: 3.4 to 5.6 semitones).

### 3.2 Comparing temporal-based pitch matching to published place-based pitch matching

Data were previously collected using the same pitch matching task with place-based pitch matches (Aronoff *et al.*, 2016). Performance was compared across the two tasks in terms of the response bias and the effects of the reference ear to determine if response bias or reference ear effects, if they exist, reflect properties of temporal-based pitch matching or if they are an artifact of the pitch matching procedures.

To compare the temporal-based pitch matching data to that based on the place-based pitch matching in Aronoff *et al.* (2016), the place-based mismatches were first converted into semitones. This was done by using the default frequency allocation for Advanced Bionics (Valencia, CA) devices, the device used in Aronoff *et al.* (2016) using a HiRes120 strategy. Because this strategy uses 15 virtual channels based on 16 physical electrodes, the lower and upper frequency for each channel was used to determine the frequencies for the two electrodes making up that virtual channel. For example, if the third virtual channel, which uses electrodes 3 and 4 to create a virtual channel, has a lower and upper frequency of 578 and 646 Hz, respectively, electrode 3 was treated as having a frequency allocation of 578 and electrode 4 as having a frequency allocation of 646. For virtual channels between electrodes 3 and 4, such as when 80% of the current was delivered to electrode 3 and 20% to electrode 4, linear interpolation was used. In this case, 592 Hz would be used as the frequency allocation.

One important difference between the place-based pitch matching and the temporal-based pitch matching data was that, for the place-based pitch matching data the reference locations and the available target locations both spanned the same range (i.e., all electrodes in the array). In contrast, for the temporal-based data, the available target AM frequencies (50–300 Hz) extended 25 Hz below and 25 Hz above the reference AM frequencies (reference frequencies: 75–275 Hz; target frequencies: 50–300 Hz). This difference could have caused differences in response patterns at the edges of the response range. As such, the place-based pitch matching data were analyzed for the more comparable reference electrodes ranging from 2.5 (equal current on electrodes 2 and 3) to 14.5 (equal current on electrodes 14 and 15) with the full array as the target range.

#### 3.2.1 Response bias

**Fig. 2.**
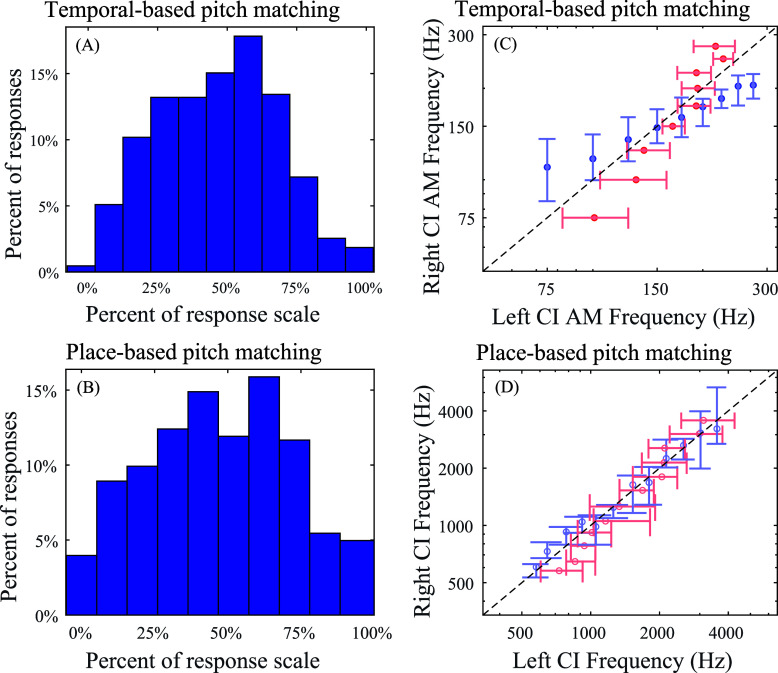
Histograms represent distributions of the responses along the response scale for the temporal-based pitch matching data (A) and the Aronoff *et al.* (2016) place-based pitch matching data (B). The 20% trimmed mean and 95% confidence interval for the target frequency for each reference frequency, pooled across participants for the temporal-based pitch matching data (C) and the previously collected place-based pitch matching data (D). Blue marks represent stimuli where the left ear was the reference. Red marks represent stimuli where the right ear was the reference.

As shown in Fig. 2(A), participants rarely chose responses at the edges of the response scale for temporal-based pitch matching. To compare this response pattern to that from the place-based pitch matching [Fig. 2(B)], the percentage of responses were split into 10 bins and the percentage of responses that were in the highest and lowest bins (i.e., the highest and lowest reported target pitches) were compared across place- and temporal-based pitch matching. This was done using a bootstrap approach, where, for each task, the same number of participants as in the original dataset were sampled with replacement and the resulting pooled responses were placed into the 10 bins shown in Figs. 2(A) and 2(B). The difference in the percentage of responses that were in the edge (highest and lowest) bins across tasks was calculated. This process was repeated 2000 times, and the confidence intervals for the difference scores were determined. The results indicated that there was a significantly higher percentage of responses in the edges of the response scale for place-based pitch matching than for temporal-based pitch matching (95% confidence interval for the difference in percentage of the data on the edges of the response scale: 1%–12%; 20% trimmed mean of the difference: 6%), suggesting a greater bias toward the center of the response space for the temporal-based pitch matching task.

#### 3.2.2 Reference ear effects

**Fig. 3.**
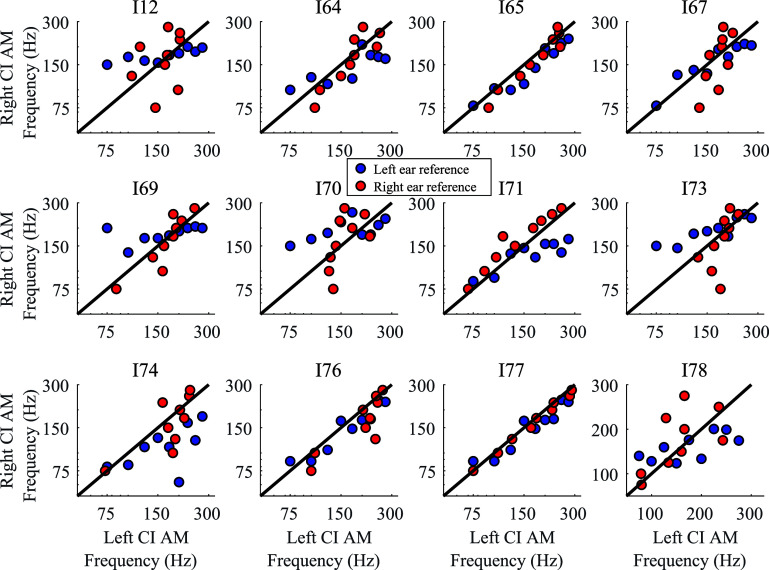
Individual results for temporal-based pitch matching. Each dot represents the median target frequency for a given reference frequency and reference ear. Blue dots represent when the left ear was the reference. Red dots represent when the right ear was the reference. Black solid line provides a reference for identical frequencies for the reference and target ear.

While the data in Aronoff *et al.* (2016) largely were collected using a single ear as a reference, some of the participants participated in the same task across multiple experiments in the laboratory where the reference ear sometimes differed within or across experiments. Across various studies in the laboratory, including unpublished data, there are datasets for five of the participants in Aronoff *et al.* (2016) with both a left and a right reference ear. As shown in Figs. 2(C) and 2(D), there is a much less pronounced effect of reference ear for the place-based pitch matches. To analyze this, slopes were calculated for each participant for each reference ear for the temporal or place-based pitch matching (no participants completed both), and the difference in the slopes for the two reference ears was compared across tasks. Because of the small sample size for the place-based pitch matching task, Yuen's test (Yuen, 1974), a robust test based on 20% trimmed means, was used instead of a bootstrap analysis. The results indicated that the slopes differed significantly more across ears for the temporal-based pitch matching task (95% confidence interval for the difference across tasks: 0.50 to 1.61 semitones; 20% trimmed mean for the difference across ears for the temporal-based pitch matching task: 0.95 semitones; 20% trimmed mean for the difference across ears for the place-based pitch matching task: –0.11 semitones). Individual participant data for temporal-based pitch matching are shown in Fig. 3.

## 4. Discussion

The results indicated that there was not a significant effect of reference frequency on mismatch magnitude. While this differs from results in other studies (Kong *et al.*, 2009; Kong and Carlyon, 2010), this may be because the reference stimuli did not extend beyond 275 Hz and the response range did not extend beyond 300 Hz. Kong *et al.* (2009) showed that rate discrimination decreased for rates above 300 Hz, although some participants were sensitive to rates as high as 900 Hz (Kong and Carlyon, 2010). While the highest-frequency AMs used in this study (300 Hz AM frequency) may have not been well encoded given that they were less than four times the 1000 Hz pulse rate, the error patterns across reference frequencies suggest that they were still perceived as high-frequency AMs given that errors for high-frequency references were not significantly larger than those for lower-frequency references.

Comparisons with previously collected pitch matching data revealed differences between results using temporal- and place-based pitch matching tasks. Participants had a notably larger bias to choose responses near the middle of the response scale with the temporal-based pitch matching task. This could be seen in the response bias analysis itself, which showed a significantly smaller percentage of responses at the edge of the response scale with the temporal-based pitch matching task. This could also be seen in the strong effect of the reference ear for the temporal-based pitch matching task, resulting in significantly larger differences in slopes across the reference ears for that task, biased toward responses near the middle of the response scale. Given that both the temporal- and place-based tasks used the same interface and similar procedures, this appears to be an inherent property of the difference in the stimuli. This may have resulted if participants attended more to place-based pitch, given that place did not differ during the temporal-based task, despite changes in the AM frequency of the stimuli. Such a bias could indicate that interaural place-based pitch differences are more salient than interaural temporal-based pitch differences for CI users, although this is not consistent with unilateral comparisons of temporal- and place-based pitch (e.g., Pijl, 1997). It also cannot be ruled out that this effect was influenced by participants completing place-based pitch matching first.

There are a number of potential limitations to this study. The use of AM high-rate pulse trains rather than unmodulated low-rate pulse trains may have decreased accuracy for the higher AM frequencies, resulting from decreased temporal sensitivity. van Hoesel (2007) found worse ITD sensitivity for higher rate temporal information, whether it was delivered through higher pulse rates or higher modulation rates. However, the detrimental effect of higher temporal information was magnified when manipulating the modulation rate instead of the pulse rate. A similar pattern was found in van Hoesel *et al.* (2009) and Goldsworthy *et al.* (2022). Additionally, participants' accuracy may have been affected by the lack of control of loudness growth for the temporal-based pitch matching stimuli. While all stimuli ranged from threshold levels to maximum comfort levels, loudness growth may have been inconsistent across ears, potentially reducing accuracy. Additionally, participants may have confused loudness changes as pitch changes, further distorting the results. Finally, the current data were collected with participants using Cochlear brand devices, while the Aronoff *et al.* (2016) data were collected with different participants using Advanced Bionics devices. As such, it is possible that differences across participants or devices may underlie some of the differences seen across tasks. Similarly, differences across device types, and especially array designs and electrode spacing in future studies, may lead to differences even when using the same pitch matching task.

The results from this study indicated that temporal-based pitch matching shows a different pattern than place-based pitch matching. With temporal-based pitch matching, there is a larger bias toward responses near the center of the response scale, which appears to, in part, reflect a bias driven by the reference ear. This suggests that it is critical to use both ears as references when conducting temporal-based pitch matching tasks with CI users. The underlying cause of this bias needs to be better understood to accurately interpret the results of temporal-based interaural pitch matching.

## Data Availability

The data that support the findings of this study are available from the corresponding author upon reasonable request.
